# Construction of Heterogeneous Aggregation-Induced Emission Microspheres with Enhanced Multi-Mode Information Encryption

**DOI:** 10.3390/molecules29245852

**Published:** 2024-12-11

**Authors:** Zhiwei Wu, Weiqin Yu, Fenghao Luo, Yue Jin, Ligou Pan, Qianjun Deng, Qing Wang, Mingguang Yu

**Affiliations:** 1 Guangdong Key Laboratory for Hydrogen Energy Technologies, Key Laboratory of Digital Decorative Materials for Building Ceramics in Guangdong Province, School of Materials and Energy, Foshan University, Foshan 528000, China; 2 School of Materials Science and Engineering, Nanchang Hangkong University, Nanchang 330063, China; 3 Laboratory of Quality & Safety Risk Assessment for Agro-Products, School of Food & Pharmaceutical Engineering, Ministry of Agriculture and Rural Affairs, Guangdong Engineering Technology Research Center of Food & Agricultural Product Safety Analysis and Testing, Zhaoqing University, Zhaoqing 526061, China

**Keywords:** AIE, heterogeneous microspheres, multi-mode information encryption, anti-counterfeiting, optical erasure

## Abstract

Traditional organic light-emitting materials hinder their anti-counterfeiting application in solid state due to their aggregation-caused quenching effect. A facile and straightforward method was reported to introduce AIE molecules into microspheres and manipulate different reaction parameters to prepare AIE microspheres with different morphologies. In this strategy, fluorescent microspheres with spherical, apple-shaped, and hemoglobin-like types were synthesized. Driven by the photocyclization and oxidation of tetraphenylethene, microspheres can be used as an aqueous fluorescence ink with erasable properties. The fluorescent patterns printed by microsphere ink on paper can be irreversibly erased by prolonged exposure to ultraviolet light (365 nm, 60 mw/cm^2^). Moreover, the multi-morphology microspheres can be further arranged for multiple-information encryption and anti-counterfeiting of barcodes and two-dimensional codes, in which double validation was carried out through fluorescence spectroscopy and laser confocal microscopy. This approach provides a new method for more reliable anti-counterfeiting and information encryption.

## 1. Introduction

Amid unceasing technological advancements and the dawn of the digital era, the proliferation of counterfeit merchandise and information breaches has become increasingly prevalent, resulting in substantial annual economic forfeitures. To counteract these issues and safeguard both commodity and informational integrity, a plethora of anti-counterfeiting and encryption technologies have been developed, including quick response (QR) codes [1], hologram [2], color transition [3,4], photoluminescent patterns [5,6], and stimuli-responsive substances [7,8,9]. Nonetheless, these methods commonly exhibit disadvantages such as susceptibility to forgery, high production expenses, intricate fabrication processes, and cumbersome detection procedures.

Fluorescent-based anti-counterfeiting and data encryption technologies [10,11,12,13,14] have emerged as a preeminent solution in this domain, owing to their inherent attributes of stealth, ease of design and implementation [15], and straightforward verifiability. The employment of fluorescent dyes in anti-counterfeiting applications ensures the verifiable authenticity of products and effectually thwarts the influx of substandard and counterfeit items into the marketplace. On the other hand, in the realm of information security, fluorescent encryption technologies afford protection by encoding sensitive data during transmission and storage, thereby forestalling unauthorized access and manipulation. Common fluorescent anti-counterfeiting and information encryption materials include traditional organic fluorescent materials [16,17,18,19], quantum dot materials [20,21,22], rare-earth coordination materials [23,24,25], and phosphorescent materials [26,27,28,29]. However, these materials often suffer from aggregation-induced bursting (ACQ) phenomenon, complex synthesis, and easy replication with simple information encryption.

In the exploration of hexaphenylthiopyrrolidine (HPS) and its derivatives, Tang [30] uncovered a remarkable property: HPS, which remains non-luminous in solution, paradoxically emits light upon aggregation. This unique phenomenon was termed “aggregation-induced luminescence” (AIE). The discovery of AIE materials solved the problem of ACQ which had been troubling for decades, and many AIE materials have been discovered one after another and have been continuously studied since then. AIE dyes have many advantages over traditional fluorescent dyes, such as excellent resistance to photobleaching in the aggregated state, the ability to adjust different molecular structures and chemical bonding to achieve different luminescence colors and emission wavelengths, and the ability to introduce different functional groups to achieve a variety of stimulatory responses [31,32,33,34,35,36,37,38]. The superior fluorescent characteristics and robust design flexibility of AIE materials herald their vast potential in the domains of anti-counterfeiting and information encryption technologies. In recent years, many AIE materials have been reported and used in the field of anti-counterfeiting and information encryption. For instance, Huang [39] and colleagues engineered a novel type of crosslinking vesicle by integrating a tetraphenylethene (TPE) moiety with acrylate groups. By modulating the quality of encapsulated Rhodamine B (RhB), they achieved a full visible spectrum of multi-color emissions through energy transfer from the polymerized TPE to RhB. Zhang [40] et al. introduced luminescent units with aggregation-induced emission (AIE) characteristics into photoresponsive hydrazone units to develop a series of novel photofluorescent photochromic AIE compounds to achieve high-resolving, rewritable, and intensity-variable light patterning systems.

Recently, a large number of smart materials have emerged and been used for anti-counterfeiting and information encryption [41,42,43,44,45,46,47,48,49,50]. Ren [51] et al. designed and developed a single-photophore fluorescent polymer, PGP (polyethyleneimine-grafted pyrene), which combines smart morphogenetic color change capability (morphing) and fluorescent switching effect (stealth). The aqueous solution is used as an erasable/developable fluorescent ink for inkjet printing, which can realize multiple “erase–develop” cycles by simple chemical processing. Wang [52] et al. developed a time-locked information encryption material based on a time-containing fluorescent hydrogel. This hydrogel can encode “time-locked” information by controlling the concentration of urea/urease and hydrochloric acid to adjust the fluorescence color between green and yellow on a time scale. However, these materials focus mainly on single-level anti-counterfeiting and information encryption, thus ignoring their potential for multi-modal anti-counterfeiting and information encryption. Since multi-mode security and information encryption are effective strategies to prevent information theft and counterfeiting [53,54,55,56,57], it is imperative to develop materials that can provide multi-mode encryption.

Hence, we propose a simple strategy to prepare AIE microspheres and use them for anti-counterfeiting and information encryption. The AIE monomer was introduced into the microspheres by a two-step dispersion polymerization method, and fluorescent microspheres with different morphologies were prepared by regulating various parameters in the experiments. The synthesized microspheres have controllable morphology and excellent fluorescence properties. Then, a variety of fluorescent inks with different compositions but consistent appearance were prepared from the microspheres with different morphologies. Multi-modal anti-counterfeiting and information encryption were carried out using the morphology and fluorescence of the microspheres and decoded by ultraviolet light and laser confocal microscopy. This multi-modal anti-counterfeiting and information encryption method greatly reduces the risk of information leakage, and the specific morphology of the microspheres also improves the difficulty of counterfeiting.

## 2. Results and Discussion

**Scheme 1:**
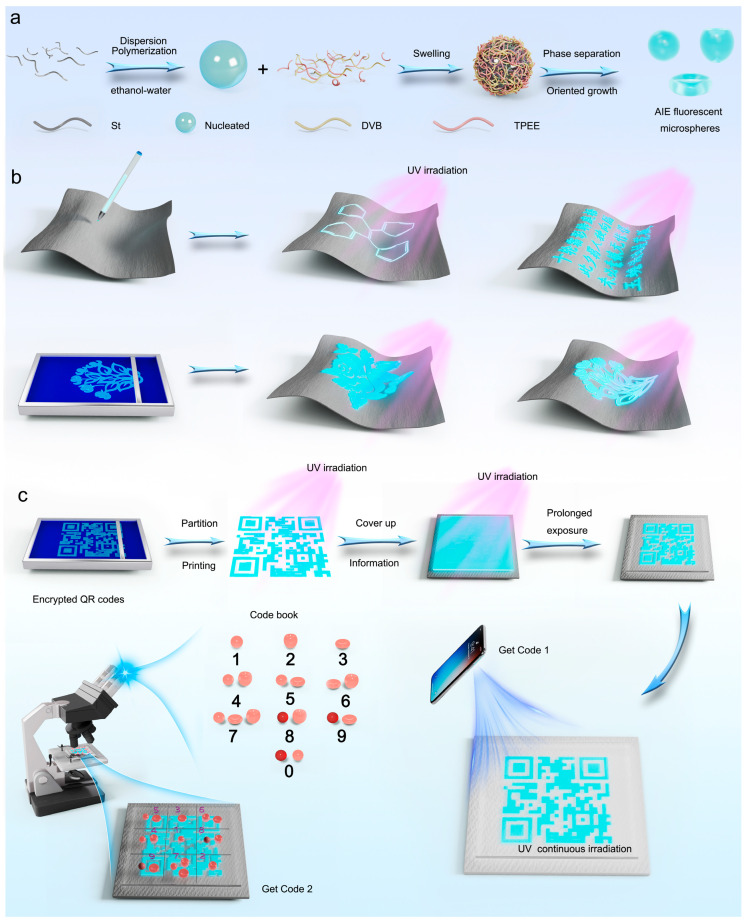
Schematic diagram of the synthetic of PS@TPEE fluorescent microspheres with different morphologies (**a**), PS@TPEE fluorescent microspheres used as inks for imaging by direct writing and screen printing to anti-counterfeiting application (**b**), PS@TPEE fluorescent microspheres with different morphologies applied to dual information encryption application (**c**), demonstration of their use as water-based anti-counterfeiting inks for information encryption.

The morphology-controlled AIE fluorescent microspheres were synthesized by two-step dispersion polymerization by using styrene (St) as a nucleating monomer, polymerizable cationic monomer 2-(methacryloyloxy)-*N*, *N*, *N*-trimethylethanaminium chloride (DMC) as the copolymer and stabilizer, 2-(4-Vinylphenyl)ethene-1,1,2-triyl)tribenzene (TPEE) as the AIE fluorescent functional monomer, and divinylbenzene (DVB) as the crosslinking agent, which was shown in Scheme 1a. The morphology of the synthesized fluorescent microspheres was controlled by changing the amount of reaction parameters, such as DMC, DVB, and TPEE.

**Figure 1:**
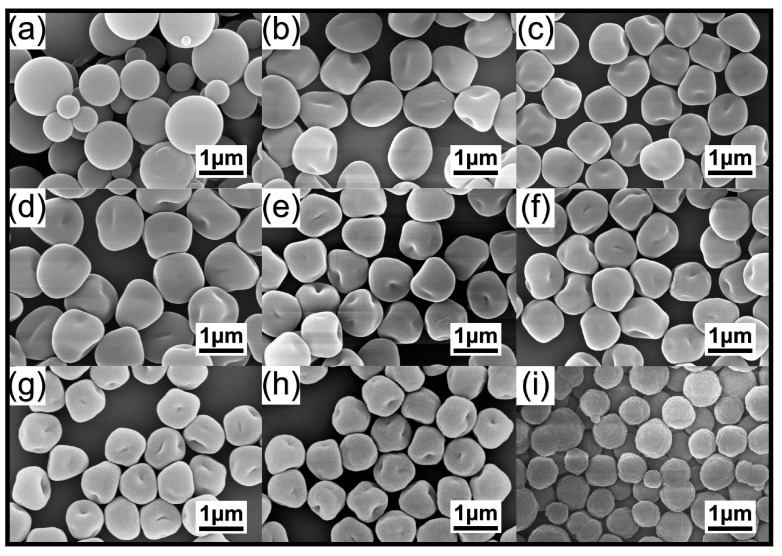
SEM images of PS@TPEE fluorescent microspheres prepared with different amounts of DMC (relative to St): (**a**) 0.025 wt%, (**b**) 0.05 wt%, (**c**) 0.1 wt%, (**d**) 0.3 wt%, (**e**) 0.5 wt%, (**f**) 1 wt%, (**g**) 1.5 wt%, (**h**) 2 wt%, (**i**) 3 wt%. Other reaction parameters: St (2.00 g), AIBN (0.04 g), TPEE (0.02 g), DVB (0.02 g), reaction medium ethanol–water (14.4 g/3.6 g, 80:20, *w*/*w*).

Figure 1 shows SEM images of poly(styrene-co-(2-(4-Vinylphenyl)ethene-1,1,2-triyl)tribenzene) (PS@TPEE) fluorescent microspheres prepared by two-step dispersion polymerization using DMC stabilizer concentrations ranging from 0.025 to 3 wt% (Table S1, entries 1–9). When the concentration of DMC was 0.025 wt%, the resulting fluorescent PS@TPEE microspheres showed obvious polydispersity, as shown in Figure 1a. This was due to the amount of stabilizer being too small, and not enough to stabilize the nucleus after nucleation, resulting in nuclear aggregation and the formation of large particles. As the amount of DMC stabilizer increased from 0.05 wt% to 2 wt%, monodispersive apple-shaped PS@TPEE fluorescent microspheres were successfully synthesized, as shown in Figure 1b–h. When DMC concentration increased to 3 wt%, PS@TPEE fluorescent microspheres obtained were polydispersed as spherical with coagulation and secondary particles, as reported by Zhang et al. [58].

The content of the crosslinker is another one of the most important parameters affecting the particle size distribution and morphology in the dispersion polymerization process. Therefore, in order to explore the effects of different amounts of DVB on the monodispersity and morphology of microspheres, a series of experiments were carried out to change the content of DVB under other constant conditions.

**Figure 2:**
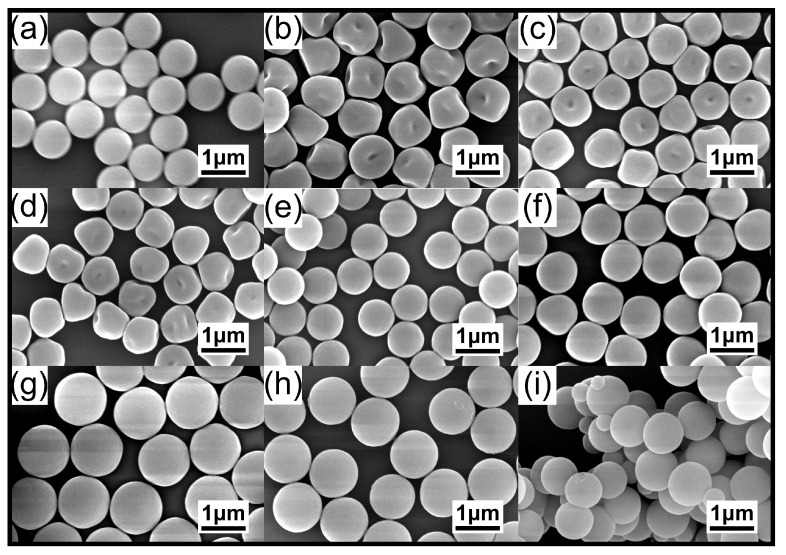
SEM images of PS@TPEE fluorescent microspheres prepared with different amounts of DVB (relative to St): (**a**) 0 wt%, (**b**) 0.5 wt%, (**c**) 1 wt%, (**d**) 2 wt%, (**e**) 3 wt%, (**f**) 5 wt%, (**g**) 8 wt%, (**h**) 10 wt%, (**i**) 12 wt%. Other reaction parameters: St (2.00 g), AIBN (0.04 g), TPEE (0.02 g), DMC (0.02 g), reaction medium ethanol–water (14.4 g/3.6 g, 80:20, *w*/*w*).

As shown in Figure 2, the pristine PS@TPEE fluorescent microspheres prepared without DVB were monodisperse and spherical. Non-spherical PS@TPEE fluorescent microspheres with apple-like morphology were obtained with the addition of DVB (Figure 2b–d). When the amount of DVB exceeded 2% relative to monomer, the PS@TPEE fluorescent became spherical again. This may be because, with the addition of crosslinking agent DVB, a crosslinked layer was formed on the surface of PS@TPEE fluorescent microspheres, and there was still a large amount of unreacted styrene monomer inside, which was a good solvent for polystyrene. Therefore, when the crosslinking agent was relatively small (0.5–2 wt% relative to styrene monomer), the crosslinked layer formed on the surface was not enough to support the internal structural collapse caused by the dissolution of polystyrene polymer to form an apple-like morphology (Figure 2b–d) [58,59]. With the further increase of DVB amount, the crosslinking density of the formed crosslinking structure increased, and the crosslinking strength was enhanced enough to maintain spherical stability, so the morphology of PS@TPEE fluorescent microspheres changed from heterogeneous structure to spherical structure again (Figure 2e–h). When the amount of DVB increased to 12 wt%, the crosslinked network on the surface of the microspheres became extremely compact, making it difficult for St to enter the interior of the particles, resulting in the secondary nucleation of the microspheres, and the latter sticking to each other and becoming polydisperse, as shown in Figure 2i. These results show that the morphology of PS@TPEE fluorescent microspheres can be changed from monodisperse and spherical to apple-shaped by changing the amount of crosslinking agent, which is a facile and versatile platform to synthesize PS@TPEE fluorescent microspheres with controlled morphologies.

**Figure 3:**
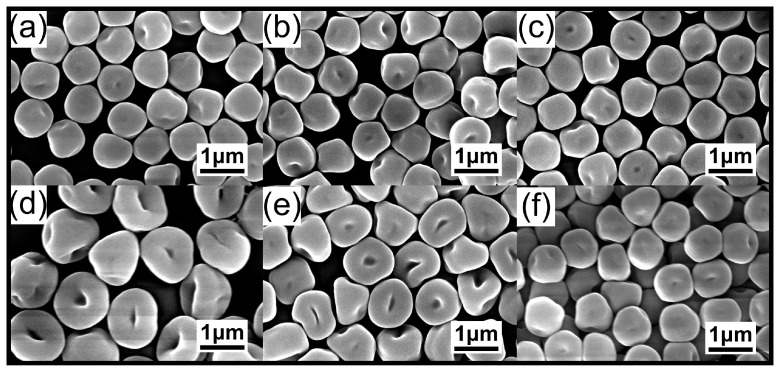
SEM images of PS@TPEE fluorescent microspheres prepared with different amounts of TPEE: (**a**) 1 wt%, (**b**) 2 wt%, (**c**) 3 wt%, (**d**) 4 wt%, (**e**) 5 wt%, (**f**) 6 wt%. Other reaction parameters: St(2.00 g), AIBN (0.04 g), DMC (0.02 g), DVB (0.02 g), reaction medium ethanol–water (14.4 g/3.6 g, 80:20, *w*/*w*).

Figure 3 shows the SEM images of the PS@TPEE fluorescent microspheres prepared with different amounts of TPEE. As shown in Figure 3, the synthesized PS@TPEE fluorescent microspheres are non-spherical with heterogeneous structures. When the amount of TPPE was 1–3 wt% of the weight of styrene monomer, the synthesized microspheres were apple-shaped. With the addition of TPEE increased from 4 wt% to 5 wt%, the morphology of the synthesized fluorescent microspheres changed to red blood cell (RBC)-like morphology. This may be due to the addition of TPEE, the critical chain length of the synthesized P(St-TPEE-DVB) polymer becoming shorter, and the rapid precipitation in the dispersion polymerization system. The precipitated P (St-TPEE-DVB) nucleus moved to the surface of the grown PS@TPEE fluorescent particles to form a crosslinked network. Unreacted monomers tend to diffuse into the PS@TPEE fluorescent particles. Therefore, the incompatibility between the monomer and the newly formed crosslinked network led to the phase separation, which resulted in the formation of RBC-like morphology [60]. When the addition of TPEE was greater than 6%, the synthesized P (St-TPEE-DVB) polymer nucleated quickly, resulting in a change in the nucleation position of P (St-TPEE-DVB), which was conducive to the formation of a uniform crosslinked network. Accordingly, the microsphere morphologies changed from RBC-like morphology to apple-shaped morphology [61].

**Figure 4:**
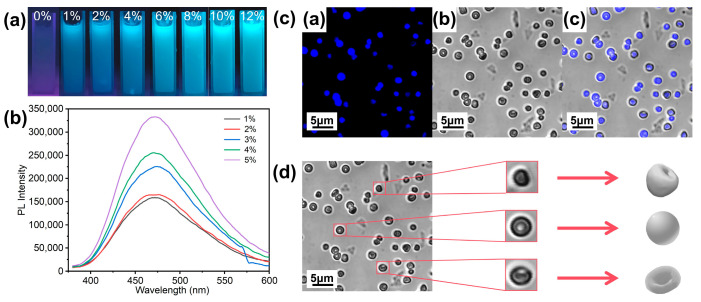
(**a**) Photo of an aqueous solution of PS@TPEE fluorescent microspheres with different amounts of TPEE under ultraviolet irradiation (The TPEE content was 0%, 1%, 2%, 4%, 6%, 8%, 10%, and 12% from left to right, λex = 365 nm). (**b**) PL spectra of PS@TPEE fluorescent microspheres at different amounts of TPEE. (**c**) Images of PS@TPEE fluorescent microspheres under a laser confocal microscope (from left to right (a–c) are darkfield fluorescence map, brightfield image, and brightfield fluorescence map). (**d**) Images of PS@TPEE fluorescent microspheres of various morphologies observed under a laser confocal microscope.

To explore the relationship between the fluorescence intensity of PS@TPEE microspheres and the content of fluorescent monomer TPEE, PS@TPEE fluorescent microsphere aqueous solutions with different TPEE contents were prepared. It can be seen that the fluorescence intensity increased gradually, as did the concentration of TPEE, with the irradiation of 365 nm UV light (Figure 4a). Figure 4b shows the fluorescence intensity curve of the PS@TPEE fluorescent microspheres in the solid state. The photoluminescence spectroscopy (PL) intensity increased with the concentration of TPEE. This was consistent with typical aggregation-induced effects and solved the problem that traditional fluorescent dyes cannot be used at high concentrations or in solid states due to the aggregative fluorescence quenching effect [62]. Therefore, PS@TPEE fluorescent microspheres with different luminescence intensities can be obtained by simply controlling the amount of TPEE.

In addition, to investigate whether the fluorescence of the synthesized fluorescence microspheres is homogeneous, laser confocal microscopy was used for observations. As shown in Figure 4c, by observing the darkfield fluorescence map, brightfield image, and brightfield fluorescence map taken by laser confocal microscopy, it can be seen that each microsphere has fluorescence, which intuitively proves the good monodispersity of the synthesized PS@TPEE fluorescent microspheres. Meanwhile, the photos taken by the laser confocal microscope can be magnified and observed, as shown in Figure 4d, which can easily identify the various morphologies of the PS@TPEE fluorescent microspheres.

**Figure 5:**
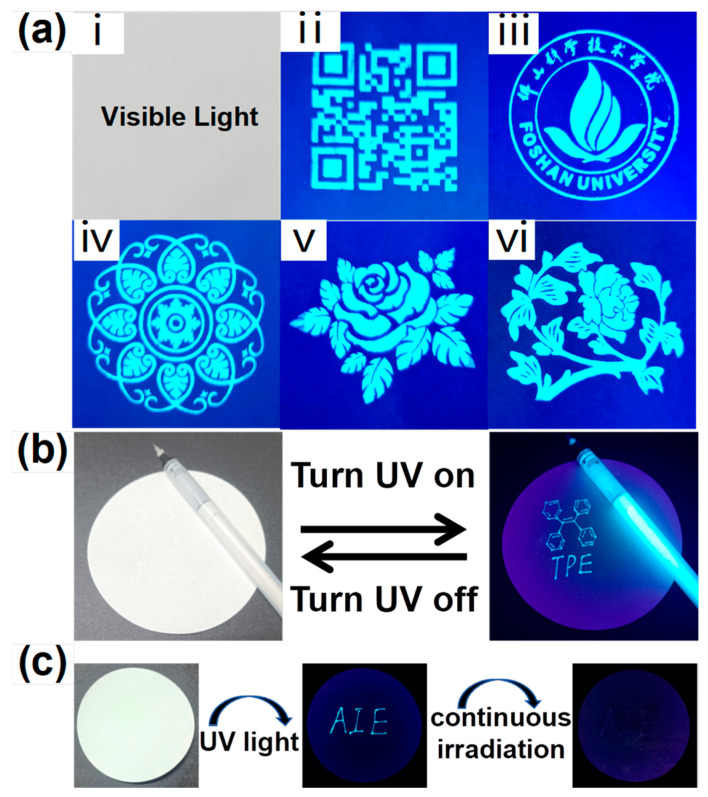
(**a**) Photo of screen-printed patterns under daylight (i) and ultraviolet (ii–vi) (PS@TPEE fluorescent microspheres are uniformly dispersed in 4 mass% polyvinyl alcohol in water). (**b**) Photograph of writing on cellulose paper with PS@TPEE fluorescent microspheres as water-based ink. (**c**) Photograph of letters written in fluorescent ink under ultraviolet light and the process of erasing.

Due to their excellent luminescent properties, PS@TPEE fluorescent microspheres can be prepared as fluorescent inks. In order to verify the application effect of the fluorescent ink, different patterns including QR codes, school logos, and flowers were printed on the paper. These patterns are invisible under visible light, but display bright blue fluorescence under ultraviolet light, as shown in Figure 4a and Movies S1 and S2. The PS@TPEE fluorescent microspheres’ (TPEE content 5%) aqueous dispersion (solid content 10 wt%) can also serve as a filler for water-based anti-counterfeiting inks to write encrypted information on paper. The written content displays clear and bright blue fluorescence under ultraviolet light, and is not visible under sunlight (Figure 5b and Movie S3). Interestingly, the prepared fluorescent ink also exhibits erasability. After prolonged exposure to ultraviolet light, TPEE derivatives undergo cyclization under ultraviolet light to form diphenylphenanthrene (DPP) derivatives with ACQ activity [63,64], causing the written letters to gradually disappear and the encrypted information to be deleted (Figure 5c). This irreversible erasure prevents encrypted information from being read multiple times, ensuring information security.

Although the inks prepared from fluorescent microspheres with different morphologies are identical in the macroscopic view, the morphologies of the microscopic compositions are different when they are enlarged. Inspired by the different microstructures of plant and animal protective colors and fluorescent microsphere inks, we explored their applications in encryption and decryption.

**Figure 6:**
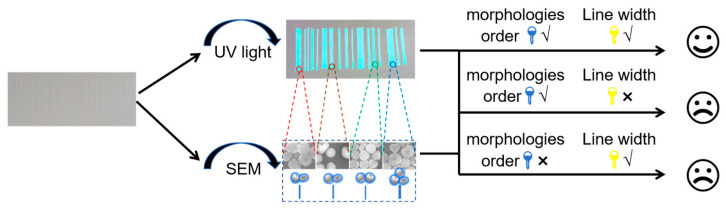
Application of barcode and information encryption prepared by PS@TPEE fluorescent microspheres with different morphologies.

Figure 6 showed barcodes printed using fluorescent inks prepared with PS@TPEE microspheres. The barcode consists of several thin lines of different thicknesses, each representing a different piece of information. We used different morphologies of PS@TPEE fluorescent microspheres to produce ink to print a barcode with fluorescence and morphology encryption. In Figure 6, the printed line width of the barcode line composed of spherical PS@TPEE microspheres was defined as “1”, the printed line width composed of apple-shaped microspheres was defined as “1.1”, the printed line width composed of hemoglobin-shaped microspheres was defined as “1.2”, and the printed line width composed of microspheres of different morphologies was the sum of their widths. As shown in the figure, the microsphere morphologies in the fluorescent barcode line were observed by SEM. From left to right, the microsphere morphologies were apple + hemoglobin type, spherical + hemoglobin type, spherical + apple type, and spherical + apple + hemoglobin type. The line widths were 2.3 (1.1 + 1.2), 2.2 (1 + 1.2), 2.1 (1 + 1.1), and 3.3 (1 + 1.1 + 1.2), respectively. By simply changing the PS@TPEE microspheres' morphologies and the corresponding line width, these printed barcode lines can be arranged and combined to obtain anti-counterfeiting patterns in the shape of microspheres that match the barcode. The order of microsphere morphologies in each line of different barcodes corresponds one-to-one with the numbers represented by the barcodes, forming a barcode database. When these barcodes were used for anti-counterfeiting labels, if the line width of the digital sequence obtained after scanning the fluorescent barcode under ultraviolet light matched the microsphere morphologies observed under the scanning electron microscope in the database, the product was genuine, otherwise it was counterfeit.

**Figure 7:**
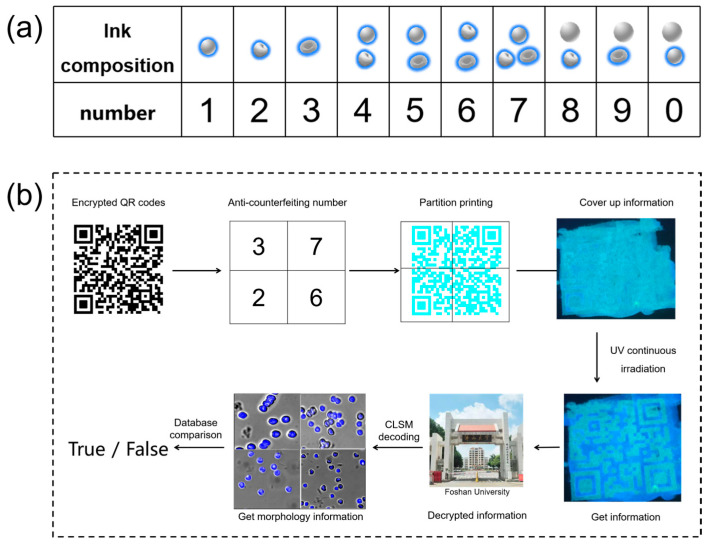
(**a**) Codebook of the corresponding numbers with different ink compositions, the spherical fluorescent microsphere in fluorescent ink indicating digital information 1, 2 for apple type, 3 for hemoglobin type, 4 for spherical + apple type, 5 for spherical + hemoglobin type, 6 for apple type + hemoglobin type, 7 for spherical + apple type + hemoglobin type, 8 for non-fluorescent microsphere + apple type, 9 for non-fluorescent microsphere + hemoglobin type, 0 for non-fluorescent microsphere + spherical fluorescent microsphere. (**b**) Process of multiple-information encryption, decryption, and information falsification of anti-counterfeiting QR code.

It is worth noting that TPE itself is a photostimuli-responsive molecule that undergoes a photooxidation reaction when exposed to UV light, causing its fluorescent color change from cyan to blue. Although polystyrene fluorescent microspheres contain a TPE structure, they do not undergo photochromic changes. This may be due to the dense and rigid structure of polystyrene microspheres preventing oxygen from entering the interior and reacting with TPE through photooxidation. Therefore, the above-mentioned fluorescent microspheres can be used for information encoding and multiple-information encryption of QR codes based on their unique structure and fluorescence characteristics, as shown in Figure 7.

Figure 7a shows a codebook of the corresponding numbers with different ink composition. The ink prepared by different morphologies of PS@TPEE fluorescent microspheres represents different digital information. For example, the spherical PS@TPEE fluorescent microsphere means digital information 1. So, ten kinds of digital information codebooks are obtained. Figure 7a shows multiple-information encryption, decryption, and information falsification processes, using different morphologies of fluorescent microsphere inks. A QR code containing product information was selected and divided into four areas, each area was printed with different morphologies of PS@TPEE fluorescent microsphere ink, and then TPE fluorescent ink was printed on the blank part of the pattern to hide the QR code. The printed QR code appeared as a cyan fluorescent color block under ultraviolet radiation, with no encrypted information. After being placed under ultraviolet light for a period of time, TPE undergoes photooxidation under the action of ultraviolet light and oxygen, and the fluorescence color changes from blue to blue, which displays the encrypted QR code (Movie S4). In this case, information about the product can be obtained by scanning the QR code using a phone, but the authenticity of the product information requires further verification of the morphology of the microspheres. The composition of the ink can be further observed by confocal laser microscopy, and the corresponding digital code can be obtained from the ink composition table and compared with the database. If the digital code corresponds to the product information, it was true, otherwise it was false. This anti-counterfeiting strategy greatly improves the security of the product, as the unique composition of the ink increases the difficulty of counterfeiting, and the ability to encode it provides another effective guarantee of product security.

## 3. Materials and Methods

### 3.1. Materials

Styrene (St, 99.5%, Aladdin, Shanghai, China) was washed with an aqueous solution of sodium hydroxide and then distilled under reduced pressure and stored in refrigeration prior to use. Vinylphenylboronic acid (97%), tetrakis(triphenylphosphine), and palladium [Pd(PPh_3_)_4_, 99%] were purchased from Innochem and used without further purification. Tetrabutylammonium tribromide (TBAB, 98%), bromotriphenylethylene (98%), potassium carbonate (K_2_CO_3_, 99%), (2-(4-Vinylphenyl)ethene-1,1,2-triyl)tribenzene (TPE, 98%), 2-(methacryloyloxy)-*N*, *N*, *N*-trimethylethanaminium chloride (DMC, 80% in water), sodium stearate (99%), and divinylbenzene (DVB, 80%) were obtained from Aladdin and used as received. Azobisisobutyronitrile (AIBN, Aldrich, 99%, Milwaukee, WI, USA) was recrystallized from ethanol and stored in a refrigerator before use. SBS (1401 YH-792) thermoplastic styrene butadiene rubber was obtained from Sinopec Baling Petrochemical Co., Ltd., (Yueyang, China) and used as received. Deionized distilled water was used in the experiments. All other reagents and solvents were purchased as analytical grade from Guangzhou Chemical Reagent Co., Ltd., (Guangzhou, China), and used without any further treatment.

#### 3.1.1. Synthesis of (2-(4-Vinylphenyl)ethene-1,1,2-triyl)tribenzene (TPEE)

TPEE was synthesized based on the published procedure [65]. In a typical procedure, bromotriphenylethylene (2.00 g, 5.97 mmol), TBAB (0.16 g), and 4-vinyl-phenylboronic acid (1.06 g, 7.16 mmol) were dissolved in toluene (50 mL). Then, 2 M potassium carbonate aqueous solution (12 mL) was added to the above solution. The reaction solution was stirred at room temperature for 30 min under nitrogen followed by adding Pd(PPh_3_)_4_ (0.005 g), and then heated to 85 °C for 24 h. After cooling to room temperature, the product was repeatedly extracted with dichloromethane. The organic layer was collected, dried with anhydrous sodium sulfate, filtered, and then rotary-distilled to obtain a crude product. Finally, the product TPEE (1.60 g, yield 90.0%) was obtained by passing through a silica gel column with *n*-hexane/CH_2_Cl_2_ (volume ratio of 5:1) as a developing agent; ^1^H NMR (400 MHz, CDCl3, Figure S1): 7.20–6.97 (m, 19H), 6.64 (dd, 1H), 5.68 (d, 1H), 5.20 (d, 1H) (Figure S1).

#### 3.1.2. Synthesis of Morphology-Controlled AIE Fluorescent Microspheres

The AIE fluorescent microspheres with controllable morphology were prepared by two-step dispersion polymerization by using polymerizable cationically charged 2-(methacryloyloxy)-*N*, *N*, *N*-trimethylethanaminium chloride (DMC) as a stabilizer. In a typical procedure, styrene (2.0 g, 10 wt% relative to the reaction system), stabilizer DMC (0.02 g, 1 wt% relative to monomer), and AIBN (0.04 g, 2 wt% relative to monomer) were dissolved in EtOH/H_2_O mixture (18.0 g, 80/20, *w*/*w*). The reaction solution was purged with nitrogen for 30 min, and then heated to 70 °C under stirring. After reacting for 1 h, different amounts of TPEE monomer and crosslinking agent DVB under N_2_ atmosphere were added to the above solution, and continued to react for 7 h. Then, the product was centrifuged, washed with ethanol/water mixture (80/20, *w*/*w*), and centrifuged repeatedly. The synthetic formulas of fluorescent microspheres with different morphologies are shown in Table S1.

#### 3.1.3. Preparation of Fluorescent Inks

Preparation of fluorescent ink based on AIE fluorescent microspheres for direct writing and screen printing.

Polyvinyl alcohol (PVA, 4.0 g, 2488), urea (1.0 g), defoamer (0.05 g, BYK-024), and sodium stearate (0.5 g) were added into a round-bottom flask containing 100 mL deionized water, and stirred to dissolve. Then, 1 mL of different kinds of microsphere dispersion with 5% solid content were added and mixed evenly to obtain fluorescent ink.

#### 3.1.4. Preparation of TPE–SBS Ink

SBS (4.0 g), urea (1.0 g), and TPE (0.05 g) were added in tetrahydrofuran (50 mL), and further gently stirred at 40 °C for 30 min to obtain the TPE–SBS fluorescent ink.

#### 3.1.5. Characterization

^1^H NMR spectra were recorded in CDCl_3_ using a NMR spectrometer (Avance NEO 400, Bruker, Karlsruhe, Germany) at a temperature of 25 °C. Morphologies of the microspheres were carried out on a scanning electron microscope (SEM) at 15 kV (Regulus8100, Hitachi, Tokyo, Japan). Samples were dispersed into ethanol with a concentration of 0.1 wt% via ultrasonication for 10 min. The dispersions were dip-cast onto the surface of the mica plate and coated with a thin layer of gold before examination. For the microspheres’ fluorescence imaging, a confocal laser scanning microscopy (CLSM, Zeiss LSM710, Carl Zeiss, Oberkochen, Germany) with an excitation wavelength of 405 nm was used. Steady-state emission and excitation spectra were measured on the steady-state/lifetime spectrofluorometer (Fluoromax-4, Horiba, Irvine, CA, USA). The fluorescence emission spectra were collected after excitation at 350 nm, and the width of each slit was maintained at 0.40 nm. All the samples were ground into powder and dried under an infrared lamp before the examination.

## 4. Conclusions

A facile and general method was presented for the synthesis of PS@TPEE fluorescent microspheres with different morphologies by two-step dispersion polymerization which can be further used as fluorescent anti-counterfeiting inks. By adjusting the concentration of DVB and TPEE, the morphology of PS@TPEE fluorescent microspheres can be transformed from spherical to apple-shaped and hemoglobin-shaped. The synthesized PS@TPEE fluorescent microspheres had an obvious AIE effect, and their fluorescence intensity gradually increased with the amount of TPEE, which avoided the ACQ effect existing in traditional fluorescent materials, and can be used as solid luminous materials. Driven by the photocycling and oxidation of tetraphene, the PS@TPEE fluorescent microspheres can be used as water-based fluorescent inks with erasable properties that can be irreversibly erased by prolonged exposure to ultraviolet light. In addition, the polymorphic PS@TPEE fluorescent microspheres can be further arranged for multiple-information encryption and anti-counterfeiting, and can be verified by fluorescence spectroscopy and laser confocal microscopy. This strategy greatly improves the difficulty of forgery and the security of information, and has a broad application prospect.

## Data Availability

The data can be obtained from the authors.

## Supplementary Materials

The following supporting information can be downloaded at: https://www.mdpi.com/article/10.3390/molecules29245852/s1, Figure S1: ^1^H NMR spectra of TPEE in CDCl_3_; Table S1: Recipes and microspheres' morphology data for two-step dispersion polymerization of St (10 wt% relative to the system) in the presence of DMC, TPEE, and DVB (relative to St); Video S1: Video of different security patterns by screen printing using polystyrene fluorescent microspheres as ink under ultraviolet light; Video S2: Video of different fluorescent pattern by screen printing using polystyrene fluorescent microspheres as ink under ultraviolet light; Video S3: Video of fluorescent numbers and letters writing by water-based ink with polystyrene fluorescent microspheres in direct writing mode under ultraviolet light; Video S4: Video of Tang poetry writing by water-based ink with polystyrene fluorescent microspheres in direct writing mode under ultraviolet light; Video S4: Video of fluorescent security two-dimensional code information gradually displayed under continuous ultraviolet light.
