# Supplementary material for: Construction of Heterogeneous Aggregation-Induced Emission Microspheres with Enhanced Multi-Mode Information Encryption
Source: Molecules. 2024 Dec 11;29(24):5852. doi: 10.3390/molecules29245852 (PMC11676549; doi:10.3390/molecules29245852)
Supplement: Supplementary file 1 [file molecules-29-05852-s001.zip › Supporting information.pdf]

Supporting information

Construction of Heterogeneous Aggregation-Induced Emission Microspheres with Enhanced Multi-Mode Information Encryption

Zhiwei Wu[1], Weiqin Yu[1], Fenghao Luo[3], Yue Jin[1], Ligou Pan[1], Qianjun Deng[1], Qing Wang[2], Mingguang Yu*[1]

[1] School of Materials and Energy, Guangdong Key Laboratory for Hydrogen Energy Technologies, Key Laboratory of digital decorative materials for building ceramics in Guangdong Province, Foshan University, Foshan 528000, China

[2] School of Food & Pharmaceutical Engineering, Zhaoqing University, Laboratory of Quality & Safety Risk Assessment for Agro-products, Ministry of Agriculture and Rural Affairs, Guangdong Engineering Technology Research Center of Food & Agricultural Product Safety Analysis and Testing, Zhaoqing 526061, China

[3] School of Materials Science and Engineering, Nanchang Hangkong University, Nanchang 330063, China

Corresponding author：Mingguang Yu

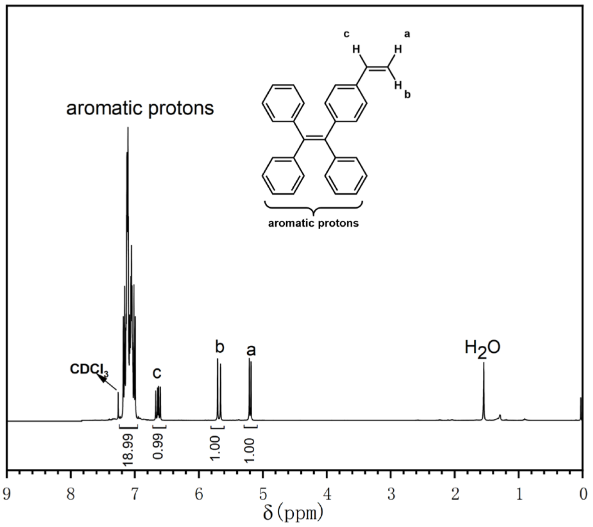

Fig.S1 ^{1}H NMR spectra of TPEE in $CDCl_3$.

Table S1 Recipes and microspheres morphologies data for two-step dispersion polymerization of St (10 wt% relative to the system) in the presence of DMC, TPEE and DVB (relative to St).

Entry	St/g	DMC wt%	TPEE wt%	DVB wt%	AIBN wt%	Ethanol/water wt%	Solid content, wt%	morphologies
1	2.00	0.025	1	1	2	80/20	10	spherical
2	2.00	0.05	1	1	2	80/20	10	apple-shaped
3	2.00	0.1	1	1	2	80/20	10	apple-shaped
4	2.00	0.3	1	1	2	80/20	10	apple-shaped
5	2.00	0.5	1	1	2	80/20	10	apple-shaped
6	2.00	1	1	1	2	80/20	10	apple-shaped
7	2.00	1.5	1	1	2	80/20	10	apple-shaped
8	2.00	2	1	1	2	80/20	10	apple-shaped
9	2.00	3	1	1	2	80/20	10	Irregular spherical
10	2.00	1	3	0	2	80/20	10	spherical
11	2.00	1	3	0.5	2	80/20	10	apple-shaped
12	2.00	1	3	1	2	80/20	10	apple-shaped
13	2.00	1	3	2	2	80/20	10	apple-shaped
14	2.00	1	3	3	2	80/20	10	spherical
15	2.00	1	3	5	2	80/20	10	spherical
16	2.00	1	3	8	2	80/20	10	spherical
17	2.00	1	3	10	2	80/20	10	spherical
18	2.00	1	3	12	2	80/20	10	spherical
19	2.00	1	1	1	2	80/20	10	apple-shaped
20	2.00	1	2	1	2	80/20	10	apple-shaped
21	2.00	1	3	1	2	80/20	10	apple-shaped
22	2.00	1	4	1	2	80/20	10	hemoglobin-shaped
23	2.00	1	5	1	2	80/20	10	hemoglobin-shaped
24	2.00	1	6	1	2	80/20	10	apple-shaped

Movie S1 Video of different security patterns by screen printing using polystyrene fluorescent microspheres as ink under ultraviolet light.

Movie S2 Video of different fluorescent pattern by screen printing using polystyrene fluorescent microspheres as ink under ultraviolet light.

Movie S3 Video of Tang poetry writing by water-based ink with polystyrene fluorescent microspheres in direct writing mode under ultraviolet light.

Movie S4 Video of fluorescent security two-dimensional code information gradually displayed under continuous ultraviolet light.
